# Sleep continuity is positively correlated with sleep duration in laboratory nighttime sleep recordings

**DOI:** 10.1371/journal.pone.0175504

**Published:** 2017-04-10

**Authors:** Akifumi Kishi, Hans P. A. Van Dongen, Benjamin H. Natelson, Amy M. Bender, Luciana O. Palombini, Lia Bittencourt, Sergio Tufik, Indu Ayappa, David M. Rapoport

**Affiliations:** 1 Division of Pulmonary, Critical Care and Sleep Medicine, Department of Medicine, New York University School of Medicine, New York, New York, United States of America; 2 Pain & Fatigue Study Center, Beth Israel Medical Center and Albert Einstein College of Medicine, New York, New York, United States of America; 3 Graduate School of Education, The University of Tokyo, Tokyo, Japan; 4 Sleep and Performance Research Center, Washington State University, Spokane, Washington, United States of America; 5 Disciplina de Medicina e Biologia do Sono, Departamento de Psicobiologia, Universidade Federal de São Paulo, São Paulo, São Paulo, Brazil; Associazione OASI Maria SS, ITALY

## Abstract

Sleep duration varies widely across individuals and appears to be trait-like. Differences in the stability of underlying sleep processes may underlie this phenomenon. To investigate underlying mechanisms, we examined the relationship between sleep duration and sleep continuity in baseline polysomnography (PSG) recordings from three independently collected datasets: 1) 134 healthy controls (ages 37 ± 13 years) from the São Paulo Epidemiologic Sleep Study, who spent one night in a sleep laboratory, 2) 21 obstructive sleep apnea (OSA) patients who were treated with continuous positive airway pressure for at least 2 months (45 ± 12 years, respiratory disturbance index <15), who spent one night in a sleep laboratory with previous experience of multiple PSG studies, and 3) 62 healthy controls (28 ± 6 years) who, as part of larger experiments, spent 2 consecutive nights in a sleep laboratory. For each dataset, we used total sleep time (TST) to separate subjects into those with shorter sleep (S-TST) and those with longer sleep (L-TST). In all three datasets, survival curves of continuous sleep segments showed greater sleep continuity in L-TST than in S-TST. Correlation analyses with TST as a continuous variable corroborated the results; and the results also held true after controlling for age. There were no significant differences in baseline waking performance and sleepiness between S-TST and L-TST. In conclusion, in both healthy controls and treated OSA patients, sleep continuity was positively correlated with sleep duration. These findings suggest that S-TST may differ from L-TST in processes underlying sleep continuity, shedding new light on mechanisms underlying individual differences in sleep duration.

## Introduction

According to the two-process model of sleep regulation [1, 2], sleep duration is regulated by the interaction of two physiological processes: a circadian process tracking time of day, and a homeostatic process tracking time awake and time asleep. The circadian process is rhythmic with a period of (approximately) 24 hours, and is driven by the circadian pacemaker in the suprachiasmatic nuclei. The homeostatic process involves a saturating exponential increase of sleep pressure during time awake, and a saturating exponential decrease of sleep pressure during time asleep. The exponential nature of the dissipation of sleep pressure implies that it dissipates faster—more “efficiently”–when it is higher.

Among normal sleepers, sleep duration varies widely across individuals in a trait-like manner [3]. Twin studies based on self-report have revealed that individual differences in sleep duration may have a genetic basis [4, 5]. Evidence from laboratory studies of sustained sleep restriction or sleep extension has suggested that being a short or long sleeper may also result from changes in the set point of the homeostatic process due to chronic prior sleep curtailment [6]. Laboratory studies have also shown physiological differences between short sleepers and long sleepers [7–10]. In particular, sleep deprivation and sleep extension studies in carefully screened “true” short sleepers and “true” long sleepers, who were operationally defined as habitually sleeping less than 6 hours per night and more than 9 hours per night, respectively, have provided evidence that “true” short sleepers live under a higher homeostatic sleep pressure than “true” long sleepers [8, 9]. The “true” short sleepers had a higher sleep efficiency (total sleep time [TST] / time in bed [TIB]) than the “true” long sleepers [8]. In the context of the two-process model of sleep regulation, the better sleep efficiency of short sleepers is compatible with the idea that short sleepers live under a higher homeostatic sleep pressure than long sleepers.

In apparent contrast, data of healthy people from a general population study in Pennsylvania [11] and from a community-based study in Hong Kong [12] not selected for extreme TST suggest that those with shorter TST may have a lower sleep efficiency than those with longer TST. Consistent with this is the finding that 24-hour norepinephrine and epinephrine levels are inversely correlated to sleep efficiency and higher in shorter sleepers than in longer sleepers [12]. Higher norepinephrine and epinephrine levels indicate greater sympathetic activity, which is associated with increased wakefulness or arousal, and may lead to less stable sleep in shorter sleepers [13–15]. Even if these disparate findings can be reconciled, it remains to be explained why short sleepers would maintain a higher homeostatic sleep pressure given that their greater sleep efficiency should allow them to reduce that pressure highly efficiently [2, 8, 9].

Analysis of dynamic aspects of sleep has elucidated novel properties of sleep regulation [16–18]. Measures of sleep continuity [19, 20], which capture the dynamics of sleep—wake transitions, have been shown to provide useful information beyond traditional sleep variables [21, 22]. For instance, survival curve (i.e., cumulative duration distribution) analysis of continuous sleep segments has revealed a difference in sleep continuity between patients with mild obstructive sleep apnea/hypopnea syndrome and controls, where traditional sleep variables such as TST and sleep efficiency did not detect any difference [20]. Other analyses of sleep—wake dynamics have shown that older people have more trouble maintaining continuous sleep (specifically non-REM sleep) compared to younger people [23, 24].

In the present study, we analyzed sleep—wake dynamics to investigate the relationship between sleep duration and sleep continuity in baseline polysomnography (PSG) records from three previously collected laboratory datasets: 1) healthy controls from the São Paulo Epidemiologic Sleep Study [25], who spent one night in a sleep laboratory, 2) obstructive sleep apnea (OSA) patients treated with and adherent to continuous positive airway pressure (CPAP) for at least 2 months [26], who spent one night in a sleep laboratory with previous experience of multiple PSG studies, and 3) healthy young adult controls who, as part of larger experiments, spent 2 consecutive nights in a sleep laboratory [3, 27–30].

## Methods

### Subjects and study protocols

Laboratory-based nocturnal PSG (NPSG) recordings and subjective and/or objective measures of sleepiness and fatigue from three previously collected datasets were examined. The three datasets were similar in that subjects were included in the analysis only when they had no known sleep pathology (except for the second group having treated OSA), no subjective sleep complaints, and normal sleep-wake schedule. For all datasets, *baseline* NPSG recordings were collected and sleep stages were scored manually according to the original [21] standard criteria or the American Academy of Sleep Medicine (AASM) [22] for the investigation of sleep. The hypnogram and age data for all subjects in all datasets are included in the S1 File.

#### Dataset 1: São Paulo controls

The subjects in this dataset were 134 subjects (68 men and 66 women, aged 37 ± 13 years [range: 20–78 years], body mass index [BMI]: 25 ± 5) free from sleep abnormalities, taken from the São Paulo Epidemiologic Sleep Study [25]. In the parent study, a three-stage cluster sampling technique with unequal selection probability was used to obtain a representative sample of the inhabitants of São Paulo, Brazil, according to gender, age (20–80 years) and socioeconomic status, excluding shift workers. For the parent cohort, a total of 1,042 subjects underwent one NPSG at the Sleep Institute in São Paulo. The study was approved by the Ethics Committee for Research of the Universidade Federal de São Paulo / Hospital São Paulo, and all subjects gave written informed consent.

From the parent study, we selected all subjects who had no discernable sleep or pulmonary disorder or complaints, that is, who met the following criteria:

1. No known diagnosis of a sleep disorder or sleep complaints, including specifically, no excessive daytime sleepiness (EDS) as evidenced by an Epworth Sleepiness Scale (ESS) [31] score ≤10, no fatigue as evidenced by a Chalder Fatigue Scale (CFS) [32] score ≤4, and no witnessed apneas reported on a sleep questionnaire [33].
2. No NPSG-assessed OSA, defined as an apnea/hypopnea index (AHI4%) < 5 [34].
3. No significant primary lung disease, as evidenced by a respiratory questionnaire validated by the American Thoracic Society [35].

#### Dataset 2: Treated OSA patients

The subjects in this dataset were 21 OSA patients (15 men and 6 women, aged 45 ± 12 years [range: 21–65 years], BMI: 38 ± 11), selected from a larger study based on compliance with CPAP for at least 2 months. The parent study involved OSA patients aged 18 years or older, prospectively recruited from all patients seen at the New York University (NYU) Sleep Disorders Center between 2006 and 2009 initially presenting with complaints of EDS and/or snoring, a NPSG showing a respiratory disturbance index (RDI; total number of apneas, hypopneas and flow-limitation events per hour of sleep) [36] >10/hour, prolonged periods of inspiratory flow limitation or REM-related and/or supine RDI >15/hour, and eligible for a clinical trial of CPAP per the treating sleep physician. Subjects who were pregnant or had medically unstable conditions, congestive heart failure, a change in medications during the trial, or history of alcohol or recreational drug abuse were excluded.

As part of the parent study, patients underwent a full-night manual laboratory CPAP titration study performed by an experienced sleep technician according to the AASM guidelines [37], where pressure was raised until all obstructive apneas, hypopneas, and runs of inspiratory flow limitation were eliminated to obtain a single, optimal pressure. Patients were given a custom CPAP machine (Fisher & Paykel HealthCare, New Zealand) with enhanced adherence monitoring capability to use at home. All patients had heated humidification but did not receive bilevel pressure, expiratory pressure relief or a pressure ramp.

Adherence to CPAP use was confirmed with downloaded data for at least 14 days after a minimum of 4 weeks of successful therapy. After an average of 9 weeks of therapy (range 6 weeks to 3 months), laboratory NPSG data were collected—these are the data used in this paper. The study was approved by NYU School of Medicine Institutional Review Board, and all patients gave written informed consent. Only those subjects with documented adherence of >4 hours/night (mean ± SD; 6 ± 1 hours) and an ESS score ≤10 on therapy were included in the present analysis.

#### Dataset 3: Healthy young adult controls

The subjects in this dataset were 62 healthy controls (24 men and 38 women, aged 28 ± 6 years [range: 22–40 years], BMI: 26 ± 5). Data were taken from *baseline* PSG measurements made in 5 different laboratory sleep deprivation experiments [3, 27–30]. In each experiment, subjects lived in a laboratory and underwent 2 successive baseline NPSG recordings prior to any other manipulations. Both baseline NPSGs were used for the present analyses.

All subjects were physically and psychologically healthy as assessed by questionnaires, physical exam and history, blood chemistry and urinalysis. They reported being good sleepers with regular bedtimes and wake-up times. Subjects were included if they had habitual sleep durations between 7 and 9 h per night [3, 27] or between 6 and 10 h per night [28–30] and habitually woke up between 06:30 and 08:30 [3, 27] or between 06:00 and 09:00 [28–30]. Habitual sleep duration and wake-up time were verified by wrist actigraphy and sleep diaries in the 7 days preceding the laboratory experiments. Subjects had no history of alcohol or drug abuse. They refrained from using drugs, alcohol, tobacco, and caffeine 7 days prior to and during the experiment and were not allowed to take any naps during this period. The Institutional Review Boards of the University of Pennsylvania and/or Washington State University approved the studies, and all subjects gave written informed consent.

### Polysomnography

#### Dataset 1: São Paulo controls

The full-night in-laboratory NPSG recordings were performed using a digital system (Embla S7000, Embla Systems, Inc., Broomfield, CO, USA). The recording protocol required subjects to abide by their usual bedtimes and wake-up times, where their schedule was verified by an actigraph and sleep diary filled out for at least 72 hours. The NPSG recordings were started between 21:30 and 02:30, and TIB was between 4.5 and 10 hours.

The following physiological variables were monitored simultaneously and continuously at a sampling rate of 256 Hz: four electroencephalogram (EEG) derivations, C3-A2, C4-A1, O1-O2, O2-A1; two electrooculogram (EOG) derivations, LOC-A2, ROC-A1; four surface electromyogram (EMG) derivations, submental, anterior tibialis muscle, masseter region, and seventh intercostal muscles; one electrocardiogram (ECG) channel, modified V2 derivation; airflow detected by oronasal thermocouple and nasal cannula pressure (50 Hz); two channels for “X-trace” belts, which record respiratory effort of the thorax and of the abdomen; channels for snoring and body position, using Embla sensors; one channel for arterial oxygen saturation (SpO_2_); and one channel for pulse rate recorded by means of a built-in Embla oximeter.

Sleep stages, arousals and leg movements were scored manually according to the AASM standard criteria for the investigation of sleep [22]. Apneas were scored and classified following the AASM 2007 recommended respiratory rules for adults [22], i.e., when there was a drop in the airflow amplitude of > 90% of baseline lasting ≥10 s. Hypopneas were scored by the “Chicago” criteria [38], i.e., when there was a discernable reduction in airflow amplitude on the nasal cannula signal lasting ≥10 s and accompanied by a decrease of ≥3% in SpO_2_ or an EEG arousal.

#### Dataset 2: Treated OSA patients

The post-treatment-initiation, in-laboratory NPSG recordings were performed using the Sandman sleep system (Embla Systems, Inc., Broomfield, CO, USA) while subjects slept wearing their usual nasal CPAP. Lights out and end of study were determined by patient report of usual sleep times. The NPSG recordings were started between 21:00 and 00:05, and TIB was between 7.9 and 11 hours.

The following physiological variables were monitored simultaneously and continuously at a sampling rate of 256 Hz: four EEG derivations, C3-M2, C4-M1, O1-M2, Fz-M2; two EOG derivations, LOC-M2, ROC-M1; chin and leg EMG; lead II ECG; rib and abdomen movement by respiratory inductance plethysmography; respiratory airflow from the CPAP analog output; and finger pulse oximetry. All sleep studies were scored manually for sleep and respiration by experienced technicians according to the AASM standard criteria for the investigation of sleep [22].

#### Dataset 3: Healthy young adult controls

The two baseline NPSG recordings were made with digital equipment (Vitaport 3, TEMEC Instruments, Kerkrade, The Netherlands or Nihon Kohden, Foothill Ranch, CA, USA). The NPSG recordings were started at 22:00 and TIB was fixed at 10 hours [28–30] or 12 hours [3, 27] depending on the experiment. For equivalence between studies in this dataset, only the first 10 hours of PSG data were analyzed for the experiments with 12 hours TIB.

The following physiological variables were monitored simultaneously at a sampling rate of 128 Hz [3] or 200 Hz [27–30]: four or six EEG derivations, C3-Ax, C4-Ax, Fz-Ax, Oz-Ax (where Ax stands for bridged mastoids) [3, 27] or C3-M2, C4-M1, Fz-M2, Oz-M1 [28] or F3-M2, F4-M1, C3-M2, C4-M1, O1-M2, O2-M1 [29, 30]; two derivations for EOG, LOC-Ax, ROC-Ax [3, 27] or E1-M2, E2-M1 [28–30]; chin EMG, submentalis; and ECG, modified lead II [3, 27] or left versus right sub-clavicle [28–30]. Sleep stages were scored manually by a registered PSG technologist according to the original [21] standard criteria [3, 27] or the new AASM [22] standard criteria [28–30] for the investigation of sleep.

### Measurement of sleepiness and fatigue

#### Dataset 1: São Paulo controls

Subjective sleepiness and fatigue were assessed using the ESS [31] and CFS [32]. ESS is an 8-item questionnaire assessing a person’s expectation of falling asleep in different circumstances. CFS is a 14-item questionnaire measuring symptoms of physical and mental fatigue. After the PSG study, subjects were asked about their quality of sleep and rated it as “better,” “the same” or “worse” than usual.

#### Dataset 2: Treated OSA patients

Subjective sleepiness was assessed using the ESS [31]. Objective sleepiness was measured with the Multiple Sleep Latency Test (MSLT) [39] and a 20-minute psychomotor vigilance test (PVT) [40]. The MSLT is a PSG-based technique involving 20-minute sessions in which sleep latency is measured as an objective index of physiological sleepiness. The PVT is a serial reaction time test that measures sustained attention as an index of behavioral alertness. These tests were administered 4 times at 2-hour intervals beginning at 09:00. Mean MSLT sleep latency and mean PVT lapses (number of reaction times > 500 ms) were calculated as the average over the 4 tests.

#### Dataset 3: Healthy young adult controls

Subjective sleepiness was assessed using the ESS [31]. Objective sleepiness was measured with a 10-minute PVT [40] administered at 2-hour intervals during scheduled wakefulness on the day after the first NPSG.

### Data analyses

#### Data reduction

Each dataset was dichotomized into shorter TSTs (S-TST) and longer TSTs (L-TST) depending on whether their TST was below or above a study-specific threshold. For dataset 1 (São Paulo controls) and dataset 2 (Treated OSA patients), in which subjects followed their usual bedtimes and wake-up times, the study-specific median TST was used as a cut point. For dataset 3 (Healthy young adult controls), in which subjects were given 10 or 12 hours TIB, TST was markedly longer, and the first tertile value of the TST distribution was used as a cut point for defining S-TST and L-TST. Additional analyses were performed using the same TST cut point, specifically the median TST of all subjects of all datasets, for all datasets with the data combined and for each dataset separately (see details in S2 File). The data from the first and second nights in dataset 3 were analyzed separately. In secondary analyses, within-subjects comparisons were made between the two NPSGs from dataset 3.

Sleep continuity was examined by generating a nonparametric survival curve of the durations of continuous sleep segments, defined as consecutive epochs scored as sleep (non-REM and/or REM) terminated by one or more epochs scored as wakefulness. Survival curves were generated for the pooled data of each dataset separately (and analyzing nights 1 and 2 of dataset 3 separately as well) using the whole-night hypnogram. The median, 25th percentile and 75th percentile durations of continuous sleep segments were also calculated for each individual subject. Survival curves were also generated for the first 3 hours of the hypnogram (datasets 1 and 3; this could not be done for dataset 2 because of insufficient number of sleep segments) to see whether results were robust to analysis timeframe. Similarly, the continuity of wakefulness during the sleep period was examined by generating a nonparametric survival curve of the durations of continuous wake segments, defined as consecutive epochs scored as wakefulness terminated by one or more epochs scored as sleep.

#### Statistical analyses

For each of the datasets separately (and for days 1 and 2 of dataset 3 separately), Mann-Whitney *U* tests were used to compare S-TST versus L-TST for PSG-assessed sleep variables, subjective and objective measures of sleepiness and fatigue, age, and BMI. For dataset 3, Wilcoxon signed-rank tests were used to compare the first night with the second night for PSG-assessed sleep variables. *χ*^*2*^ tests for independence were used to compare S-TST versus L-TST with regard to the distribution of males and females, and in dataset 1 for the question about subjective sleep quality (comparison to “usual”). Generalized Wilcoxon tests [41] were used to compare S-TST versus L-TST on the survival curves. Spearman’s *ρ* was used to examine relationships between TST as a continuous variable and median duration of continuous sleep segments—both across all datasets (controlling for study and age) and in each dataset separately. To investigate the stability of categorizing subjects as S-TST or L-TST based on TST in dataset 3 (where two consecutive nights were recorded), we calculated the intraclass correlation coefficient (ICC) [42] for TST. We also performed this analysis for median duration of continuous sleep segments. The ICC was derived from a mixed-effects analysis of variance (ANOVA) with a random effect on the intercept and covariates for night number and study.

## Results

### Demographics and traditional sleep variables

Table 1 compares age, sex, BMI, and traditional sleep variables between the S-TST and L-TST groups. Within each dataset, age, sex and BMI did not differ significantly between the two groups.

**Table 1:** Characteristics of subjects, sleep variables, sleep continuity and measures of sleepiness and fatigue for all datasets.

	Dataset 1		Dataset 2		Dataset 3			
					1st night		2nd night	
	S-TST	L-TST	S-TST	L-TST	S-TST	L-TST	S-TST	L-TST
**Number of subjects**	67	67	10	11	21	41	21	41
**% Men**	47.8	53.7	80.0	63.6	38.1	39.0	23.8	46.3
**Age (years)**	39.1 ± 12.7	34.8 ± 12.8	50.2 ± 9.8	41.0 ± 13.4	26.6 ± 4.1	29.5 ± 6.0	27.8 ± 5.9	28.1 ± 5.3
**Body mass index (kg/m**^**2**^**)**	25.6 ± 5.8	24.4 ± 4.1	35.5 ± 10.9	40.3 ± 11.4	25.6 ± 4.5	24.7 ± 3.5	27.5 ± 4.0	24.7 ± 4.4
***Sleep structure***								
**Time in Bed (min)**	355.9 ± 48.7	434.5 ± 63.0**	521.3 ± 31.8	538.9 ± 44.5	599.8 ± 0.4	599.8 ± 0.5	599.8 ± 0.7	599.8 ± 0.7
**Total Sleep Time (min)**	270.2 ± 56.1^‡^	378.0 ± 45.2^‡^	382.3 ± 89.7^‡^	477.2 ± 28.8^‡^	460.6 ± 38.7^‡^	544.0 ± 19.5^‡^	459.9 ± 42.7^‡^	540.5 ± 17.2^‡^
**Sleep Efficiency (%)**	76.7 ± 16.5	88.2 ± 8.1**	73.8 ± 18.4	88.9 ± 6.5**	76.8 ± 6.4	90.7 ± 3.3**	76.7 ± 7.1	90.1 ± 2.9**
**Wakefulness After Sleep Onset (%)**	16.3 [8.8–23.5]	10.2 [4.4–14.1]**	14.9 [13.2–22.3]	6.3 [4.6–9.4]**	15.2 [12.0–20.5]	6.1 [3.7–9.6]**	16.0 [8.5–21.0]	5.9 [4.0–8.2]**
**N1 (%)**	3.2 [2.2–5.2]	2.6 [1.6–4.1]	12.1 [10.7–13.5]	11.3 [9.7–14.0]	4.5 [3.6–5.9]	4.5 [3.4–5.9]	4.8 [3.4–6.5]	4.0 [3.0–6.0]
**N2 (%)**	42.9 [37.4–49.6]	47.7 [42.0–51.9]**	49.3 [46.1–51.1]	52.5 [48.4–59.4]	44.0 [39.5–47.3]	51.2 [45.4–54.8]**	43.2 [39.3–50.6]	51.4 [46.8–54.4]**
**N3 (%)**	21.0 [14.3–24.3]	20.1 [16.2–24.8]	3.9 [0.6–7.4]	4.6 [0.9–13.9]	16.4 [12.8–18.8]	14.7 [10.8–17.0]	13.1 [8.6–18.1]	14.3 [11.6–15.9]
**REM Sleep (%)**	14.2 [10.5–16.4]	17.4 [12.7–21.1]**	16.5 [12.6–21.5]	21.8 [14.9–23.0]	20.1 [16.4–23.4]	23.7 [20.3–26.1]**	20.3 [16.6–22.5]	23.7 [21.9–26.5]**
**Sleep Latency (min)**	11.5 [5.5–24.0]	8.0 [4.0–18.4]	9.3 [7.0–29.0]	15.0 [9.9–21.3]	32.5 [16.9–47.1]	14.0 [10.4–20.6]**	41.0 [24.4–69.8]	18.5 [13.8–32.0]**
**REM Latency (min)**	96.5 [72.4–140.6]	87.5 [65.8–111.8]	125.3 [77.0–150.0]	84.0 [65.3–161.6]	83.5 [69.5–144.3]	67.0 [60.4–82.0]*	70.0 [60.5–93.3]	72.5 [60.9–84.1]
**Number of Awakenings**	20.0 [13.0–25.0]	20.0 [15.5–28.0]	29.0 [19.8–34.5]	19.0 [17.0–33.0]	29.0 [21.0–33.0]	25.0 [19.0–34.0]	28.0 [23.0–30.0]	27.0 [23.0–36.0]
***Sleep continuity***								
**25th percentile for duration of continuous sleep segments (min)**	1.5 [1.0–3.1]	2.3 [1.3–5.9]	1.1 [0.8–1.5]	1.5 [1.0–5.5]	2.5 [1.0–3.9]	4.5 [2.5–8.2]**	2.8 [1.9–4.3]	4.3 [2.8–7.0]*
**Median duration of continuous sleep segments (min)**	6.0 [2.5–11.5]	10.5 [5.6–17.4]*	5.3 [4.5–12.8]	9.0 [4.4–20.3]	9.5 [5.5–11.9]	14.5 [9.5–20.6]**	9.5 [7.4–12.3]	12.0 [8.8–15.1]^†^
**75th percentile for duration of continuous sleep segments (min)**	19.6 [10.8–30.8]	29.6 [19.8–37.2]*	19.9 [16.0–41.5]	28.0 [18.4–47.8]	21.0 [15.4–29.4]	28.3 [22.1–41.8]**	24.8 [18.1–29.2]	25.5 [19.9–37.9]
***Sleepiness and fatigue***								
**ESS**	5.0 [3.0–7.0]	5.0 [3.0–7.0]	6.5 [5.0–8.0]	6.0 [3.3–9.5]	6.0 [3.8–7.3]	5.0 [3.0–6.3]	5.0 [3.0–7.0]	5.0 [3.0–7.0]
**CFS**	1.0 [0.0–3.0]	2.0 [0.0–3.0]						
**PVT lapses**			8.8 [5.4–12.4]	9.0 [4.3–12.6]	0.8 [0.5–1.5]	0.5 [0.2–0.9]	0.7 [0.2–1.5]	0.5 [0.2–1.2]
**MSLT (min)**			2.5 [1.0–4.3]	2.3 [1.7–11.6]				

Values are means ± SD or medians [25th percentile– 75th percentile].

**P<0.01,

*P<0.05,

^†^*P* = 0.06 for difference between S-TST and L-TST.

^‡^No statistical tests were performed for TST as groups were selected on the basis of TST.

**Fig 1:**
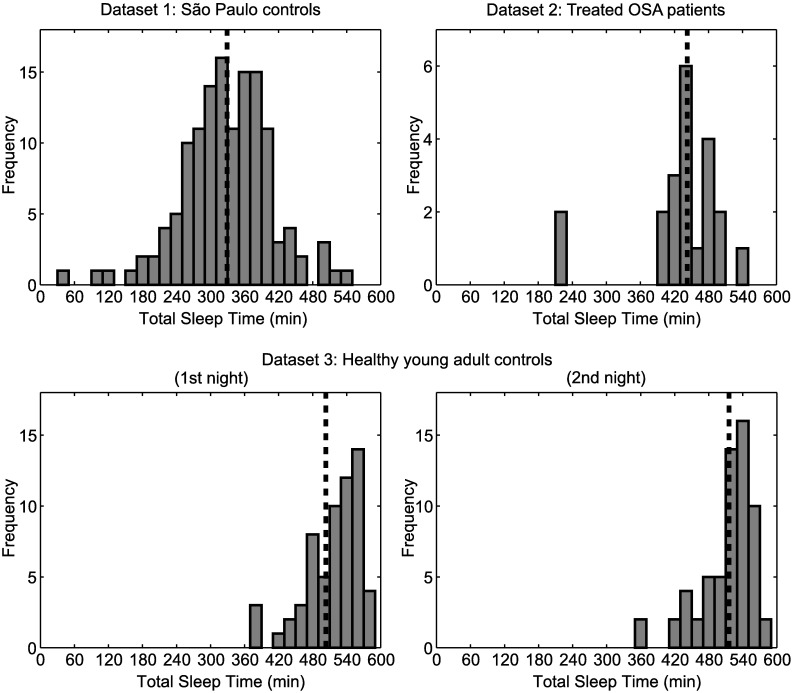
Distribution of total sleep time in each dataset. Distribution of total sleep time (TST) for dataset 1 (São Paulo controls), dataset 2 (Treated OSA patients) and the 1st and the 2nd nights of the dataset 3 (Healthy young adult controls). Dashed lines represent the threshold values used to separate subjects into those with shorter TST and those with longer TST in each dataset.

The distribution of TST in each dataset is shown in Fig 1. The medians of TST for dataset 1 (São Paulo controls) and for dataset 2 (Treated OSA patients) were 334.3 min and 442.5 min, respectively. For dataset 3 (Healthy young adult controls), the first tertile values for the first and the second nights were 503.3 min and 515.6 min, respectively. These values were used to separate subjects into S-TST and L-TST groups for each night. For dataset 3, 68% of subjects remained in the same group across the two nights of PSG (note that our analyses were not contingent on stability of group assignment).

For all datasets (and for both nights of dataset 3), sleep efficiency was significantly less in S-TST than in L-TST, and percentage of wakefulness after sleep onset (WASO) was significantly greater in S-TST than in L-TST (Table 1). For dataset 1 and for both nights of dataset 3, percentages of N2 and REM sleep were significantly less in S-TST than in L-TST. For both nights of dataset 3, sleep latency was significantly longer in S-TST than in L-TST. For the first night only, REM latency was significantly longer in S-TST than in L-TST. Taken together, these results suggest that sleep architecture was different between S-TST and L-TST and, notably, that sleep was more consolidated in L-TST than in S-TST. For dataset 3, none of the sleep variables differed significantly between the first and second nights.

These results held up in additional analyses using the same TST cutoff for all datasets, with the data combined but statistically controlling for dataset and age (or age alone), as well as analyzing each dataset separately (see S2 File).

### Continuity of sleep and wake

Comparisons of median, 25th percentile and 75th percentile durations of continuous sleep segments between S-TST and L-TST groups in each dataset are shown in Table 1. For dataset 1 (São Paulo controls) and the first night of dataset 3 (Healthy young adult controls), median, 25th percentile and 75th percentile durations of continuous sleep segments were significantly shorter in S-TST than in L-TST (Table 1). For the second night of dataset 3, 25th percentile duration of continuous sleep segments was significantly shorter in S-TST than in L-TST and median duration of continuous sleep segments showed a trend to being shorter in S-TST than in L-TST, while 75th percentile duration of continuous sleep segments did not differ significantly between S-TST and L-TST. There were no significant differences in dataset 2 (Treated OSA patients).

**Fig 2:**
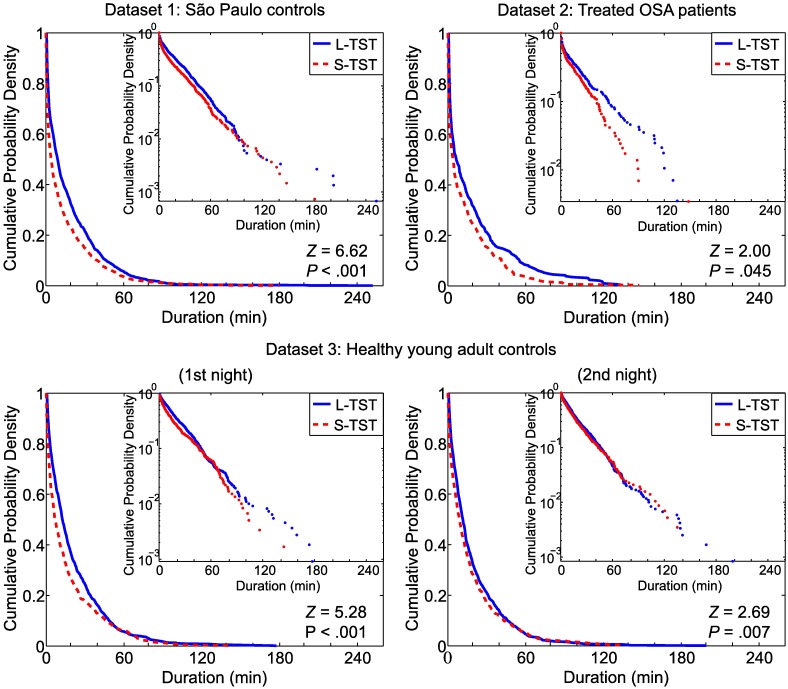
Continuity of sleep for whole-night hypnograms in each dataset. Survival curves (cumulative probability distributions) of continuous sleep segments in the S-TST (dashed, red) and L-TST (solid, blue) groups for whole-night hypnograms, for dataset 1 (São Paulo controls), dataset 2 (Treated OSA patients) and the 1st and the 2nd nights of the dataset 3 (Healthy young adult controls) are shown. Insets: Same but plotted semilogarithmically. The S-TST group consistently showed a significant shift toward shorter bouts of sleep compared with the L-TST group.

**Fig 3:**
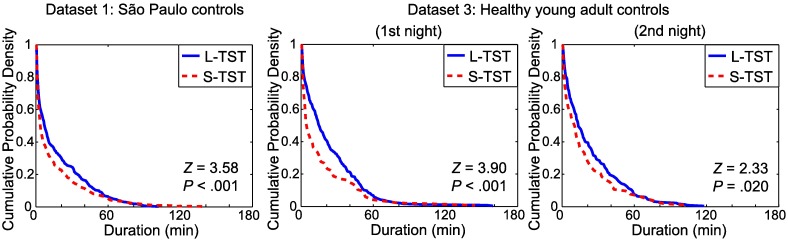
Continuity of sleep for the first 3 hours of the hypnograms in each dataset. Survival curves (cumulative probability distributions) of continuous sleep segments in the S-TST (dashed, red) and L-TST groups (solid, blue) for the first 3 hours of the hypnograms, for dataset 1 (São Paulo controls) and the 1st and the 2nd nights of the dataset 3 (Healthy young adult controls) are shown. The S-TST group consistently showed a significant shift toward shorter bouts of sleep compared with the L-TST group.

Survival curves (cumulative duration distributions) of continuous sleep segments in the whole night hypnograms of S-TST and L-TST are shown in Fig 2. For all datasets (and both nights of dataset 3), there were significant shifts toward shorter (i.e., less continuous) bouts of sleep in S-TST than in L-TST (Fig 2). This finding remained for dataset 1 and for both nights of dataset 3 when restricting the analysis to the first 3 hours of the hypnograms (Fig 3).

**Fig 4:**
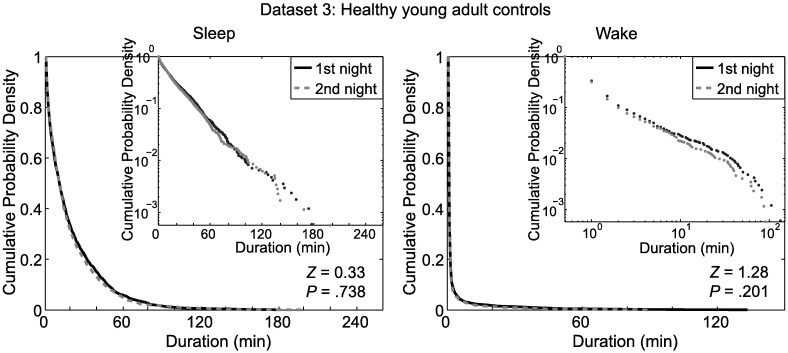
Continuity of sleep and wake of the first and the second nights in dataset 3. Survival curves (cumulative probability distributions) of continuous sleep segments (left) and continuous wake segments (right) for the whole-night hypnograms of the first night (solid, black) and the second night (dashed, gray) in dataset 3 (Healthy young adult controls) are shown. Insets: Same but plotted semi-logarithmically (left) and double-logarithmically (right). In both panels, survival curves did not differ significantly between the first and second nights.

For each dataset, the distributions of the durations of continuous sleep segments in the whole night hypnograms appeared to be exponential, as evidenced by the linearity of the survival curves when drawn in semi-logarithmic plots (Fig 2, insets). For dataset 3, the survival curves of continuous sleep segments for whole night hypnograms did not differ significantly between the first and second nights (Fig 4).

**Fig 5:**
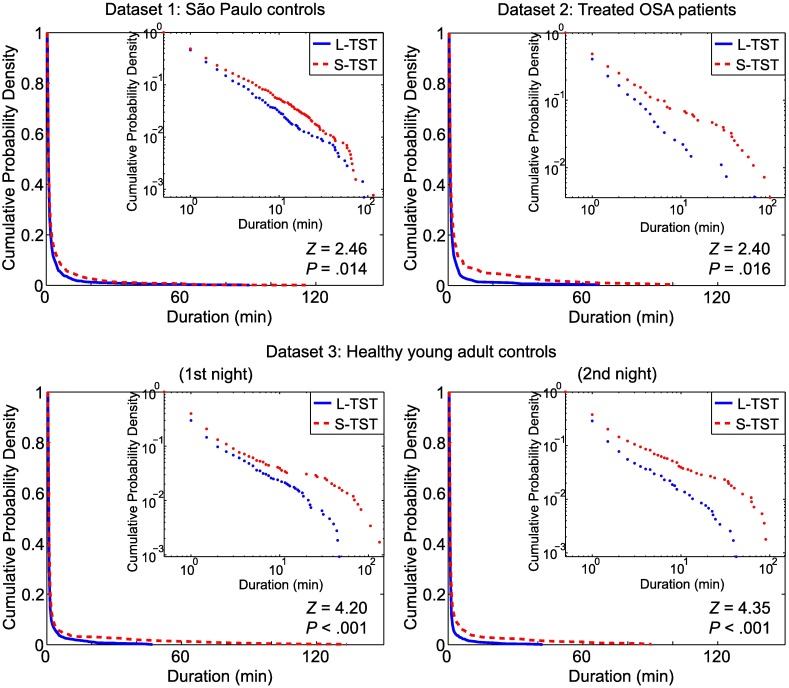
Continuity of wake for whole-night hypnograms in each dataset. Survival curves (cumulative probability distributions) of continuous wake segments in the S-TST (dashed, red) and L-TST (solid, blue) groups for whole-night hypnograms, for dataset 1 (São Paulo controls), dataset 2 (Treated OSA patients) and the 1st and the 2nd nights of the dataset 3 (Healthy young adult controls) are shown. Insets: Same but plotted double-logarithmically. The S-TST group consistently showed a significant shift toward longer bouts of wakefulness compared with the L-TST group.

Survival curves of continuous wake segments in the whole night hypnograms of S-TST and L-TST are shown in Fig 5. For all datasets (and for both nights of dataset 3), there were significant shifts toward longer (i.e., more continuous) bouts of wakefulness in S-TST than in L-TST. For each dataset, the durations of continuous wake segments appeared to exhibit a power-law (or multi-exponential [43]) distribution, as evidenced by the linearity of the survival curves when drawn in double-logarithmic plots (Fig 5, insets). For dataset 3, the survival curves of continuous wake segments did not differ significantly between the first and second nights (Fig 4).

These results held up in a follow-up, bootstrap-based analysis that accounted for the number of segments contributed by each subject (see S3 File). Also, the results held up in additional analyses using the same TST cutoff for all datasets, with the data combined but statistically controlling for dataset and age (or age alone), as well as analyzing each dataset separately (see S2 File).

### Correlation between total sleep time and continuity of sleep

**Fig 6:**
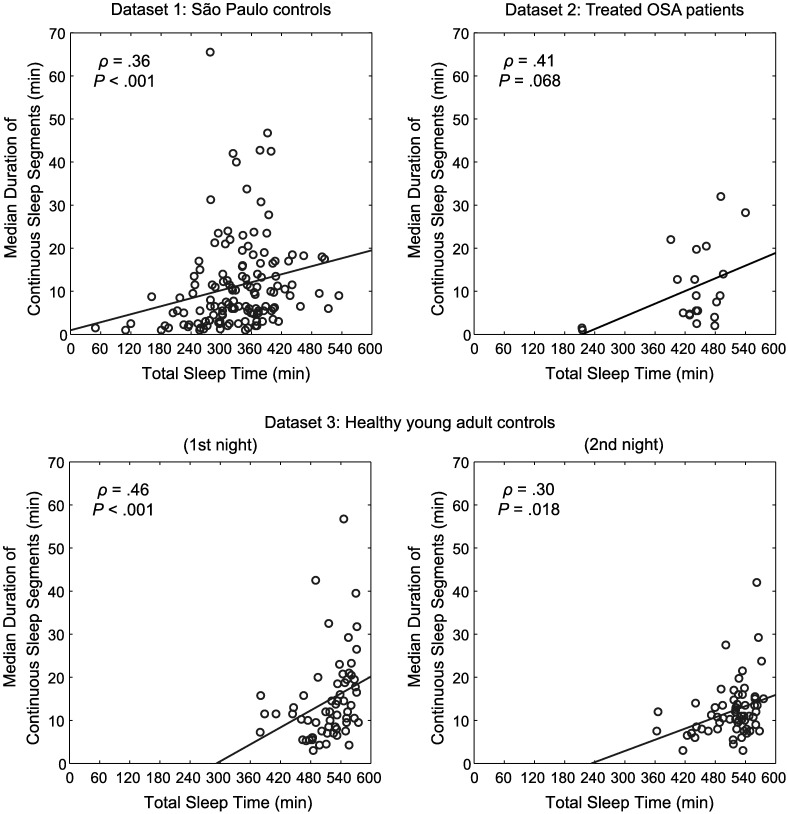
Correlation between total sleep time and continuity of sleep. Scatter plots of the relationship between TST and median duration of continuous sleep segments for dataset 1 (São Paulo controls), dataset 2 (Treated OSA patients) and the 1st and the 2nd nights of the dataset 3 (Healthy young adult controls) are shown. Correlations were significant and positive for dataset 1 and both nights of dataset 3 and tended to significance for dataset 2.

The relationship between TST and the median duration of continuous sleep segments is shown in Fig 6. Across all datasets, and controlling for study, there was a significant positive correlation between TST and median duration of continuous sleep segments (*ρ* = 0.35, *P*<0.001). This held true for analyses controlling for age (*ρ* = 0.28, *P*<0.001), for analyses using either the first or the second night of dataset 3 (*ρ* = 0.35, *P*<0.001 and *ρ* = 0.34, *P*<0.001, respectively) as well as for the dataset excluding subjects whose TST was less than 4 hours (*ρ* = 0.26, *P* = 0.004). Significant correlations were also found for dataset 1 and the both nights of dataset 3 when the datasets (and the two nights of dataset 3) were analyzed separately; there was a trend for dataset 2, which has relatively small sample size (Fig 6).

### Intraclass correlation coefficients

Estimates for between-subjects and within-subjects variance and the ICC values derived from them for the two NPSGs of dataset 3 (Healthy young adult controls) are shown in Table 2. Using published benchmarks [44], the ICC values were ‘moderate’ for TST and median duration of continuous sleep segments. Thus, individual differences in key variables describing sleep duration and sleep continuity were moderately stable. For TST, ICC values have been published previously [3]; the previously reported values are very similar to those found presently.

**Table 2:** Intraclass correlation coefficients of sleep duration and median duration of sleep segments for dataset 3.

	VAR_bs_	VAR_ws_	ICC (95% CI)
**Total sleep time (min)**	1137.9 ± 327.0	1061.6 ± 192.2	0.52 (0.31–0.68)
**Median duration of continuous sleep segments (min)**	37.3 ± 10.6	33.8 ± 6.1	0.52 (0.32–0.68)

Values are means ± SD. VAR_bs_, between-subjects variance; VAR_ws_, within-subjects variance; CI, confidence interval.

### Measures of sleepiness and fatigue

For the three datasets, none of the subjective measures of sleepiness and fatigue (ESS and CFS scores) nor the objective measures of sleepiness (PVT lapses and mean MSLT) differed significantly between the S-TST and L-TST groups (Table 1).

For dataset 1 (São Paulo controls), the distribution of subjective sleep quality ratings did not differ significantly between groups. The percentage of subjects who reported their recorded sleep as “better” or “the same” compared to their usual sleep was 72.1% for S-TST and 76.6% for L-TST. The percentage of subjects who reported their recorded sleep as “worse” than their usual sleep was 27.9% for S-TST and 23.4% for L-TST.

## Discussion

### Interpretation of findings

We found that sleep continuity is positively correlated with objective sleep duration (TST) in healthy controls as well as treated OSA patients. Across a wide range of naturally occurring sleep durations in normal and corrected-to-normal (i.e., CPAP-treated) sleepers recorded polysomnographically in the laboratory, S-TST displayed less sleep continuity than L-TST, as evidenced by lower sleep efficiency, greater percentage of WASO and systematic differences in survival curves indicating shorter continuous sleep segments. While systematic differences between S-TST and L-TST in recent sleep history cannot be ruled out completely, no differences in baseline waking performance and sleepiness were observed. Taken together, these results suggest that the S-TST phenotype may be due, at least in part, to a lower neurobiological ability of conserving sleep continuity.

Our findings are consistent with a genetic explanation of natural sleep duration as indicated by twin studies [4, 5], but raise questions about the role of sleep insufficiency and/or prevailing sleep homeostatic pressure—suggesting instead an underlying physiological difference between S-TST and L-TST in the stability of sleep processes. This would be consistent with a previous study [12] showing S-TST had lower sleep efficiency and increased 24-hour urinary norepinephrine and epinephrine levels compared to L-TST. The higher sympathetic activity indicated by increased catecholamine levels is consistent with increased arousal, which could in turn lead to reduced stability of sleep processes, thereby producing the S-TST phenotype.

A natural association between sleep duration and sleep continuity exists in healthy subjects sleeping after acute total sleep deprivation; these individuals have longer and more continuous sleep than during baseline sleep [3, 45]. Such a relationship is also seen within and between subjects in normal aging—sleep in older people tends to be shorter and more fragmented than in younger people [23, 24, 45, 46]. However, since there was no significant age difference between S-TST and L-TST subjects studied here (Table 1), the relationship between sleep duration and sleep continuity appeared to exist intrinsically. This is corroborated by the relative stability of inter-individual differences in both TST and median duration of continuous sleep segments across the two nights of dataset 3 (Healthy young adult controls), as indicated by moderate ICC values (Table 2).

It could thus be that S-TST may have less homeostatic sleep pressure than L-TST. However, this conclusion seems incongruent with the idea that S-TST have greater sleep efficiency which allows them to live under higher homeostatic sleep pressure, as has been shown in seminal studies of specifically selected “true” extremely short and long sleepers [8, 9]. Similar to our finding for the whole night, S-TST in our datasets (1 and 3) did not have greater sleep continuity in the first 3 hours of sleep (Fig 3). These results make the explanation of greater sleep efficiency in the S-TST unconvincing.

That does not necessarily mean, however, that individual differences in sleep efficiency are entirely irrelevant in our datasets. Mechanisms underlying the existence of individual differences in sleep duration may well be multi-factorial, and which factor(s) dominate may depend on sample selection criteria. Thus, individual differences in sleep efficiency in earlier studies may have reflected selection bias favoring only subjects with extreme S-TST and L-TST [7–9]; such a bias did not exist in the present study where subjects were not pre-selected on the basis of sleep duration.

Reconciliation of the seemingly competing views on the nature of S-TST and L-TST is possible with dynamic modeling. Lo and colleagues [19] introduced a dynamic stochastic model for sleep—wake transitions in which the duration of wake bouts was simulated according to a power-law distribution and the duration of sleep bouts was modeled according to an exponential distribution. These distributions were evident in our datasets (Figs 2 and 5). In the model, sleep–wake state evolves over time based on a random-walk process as derived from interaction between sleep-promoting and wake-promoting neuronal populations [47, 48]. In this framework, our findings indicate that the waking pressure on the random-walk process towards sleep is smaller in S-TST than in L-TST. In addition, S-TST need to traverse a smaller distance in the random walk process during sleep before wakefulness is triggered. However, in the model this distance in the random walk process during sleep does not necessarily affect sleep depth, and in our data deep sleep (stage N3) did not differ between S-TST and L-TST. In the context of the model, these properties are indicative of traits for overall lower homeostatic sleep pressure and less sleep need in S-TST compared to L-TST.

### Potential confounds

Various possible confounds need to be considered when interpreting our findings. These range from the impact of outliers to first night effects, environmental factors and circadian timing. However, several aspects of our experimental conditions and observations make it unlikely that these factors were influential.

In each of the three datasets, we included only subjects with no known sleep pathology. Nonetheless, in dataset 1 (São Paulo controls), some of the subjects exhibited extremely short sleep duration. It would be possible that these cases skewed our findings. We repeated the analyses after excluding subjects whose TST values were less than 4 hours, and found that the positive correlation between sleep duration and sleep continuity persisted, indicating that our results were not confounded by outliers. Also, the median duration of continuous sleep segments (Fig 6) was comparable to that of previously published data of healthy subjects [19], suggesting that our data of sleep continuity are within the normal range. Additionally, none of the subjects in dataset 1 took any naps prior to the study, such that short nighttime sleep duration could not have been caused by reduced homeostatic sleep pressure due to prior napping.

In principle, a first night effect could have affected our results. Dataset 3 (Healthy young adult controls) allowed us to rule this out, as we found no significant differences between the first and second nights in the sleep laboratory (Table 1 and Fig 4). Further, the subjects in dataset 2 (Treated OSA patients) had already undergone multiple PSG studies previously, making a first night effect similarly unlikely. In dataset 1 (São Paulo controls), subjects did not undergo multiple PSG studies, but S-TST and L-TST subjects did not differentially report their subjective sleep quality during the PSG night to be better or worse than usual. These data support that our results were not confounded by a first night effect.

It is possible that the timing and duration of the laboratory sleep opportunities were different from what subjects experienced in their normal lives. Sleep opportunities in our three datasets varied considerably, with dataset 1 providing a wide range of sleep opportunities (between 4.5 and 10 hours TIB, starting between 21:30 and 02:30); dataset 2 providing a more narrow range of sleep opportunities (between 7.9 and 11 hours TIB, starting between 21:00 and 00:05); and dataset 3 providing ample sleep opportunity as part of the underlying study’s design (at least 10 hours TIB each night, starting at 22:00). Importantly, the subjects in datasets 1 and 2 were required to abide by their habitual bedtimes and wake-up times (which were verified by actigraphy at least 3 days before study or self-report), and the subjects in dataset 3 had ample TIB which should have accommodated their habitual sleep duration (habitual sleep duration was verified by self-report and also by actigraphy in the week before study). Since the findings of our study were consistent among the three datasets, any explanation involving confounds owing to the timing of the laboratory sleep opportunities therefore does not seem viable.

Lastly, subjects’ psychological status (e.g., anxiety) or extrinsic factors such as environmental disturbances (e.g., noise) could have affected our observations. However, our studies were performed in controlled laboratory settings, and there were no differences between S-TST and L-TST in subjective sleep quality in dataset 1 or PVT lapses in datasets 2 and 3. Moreover, the consistency of results across the two nights in dataset 3 argues against the involvement of extraneous, non-sleep physiological factors.

### Limitations

As is true for all studies of S-TST and L-TST that are based on laboratory PSG, it is possible that subjects displayed different sleep characteristics in the laboratory than they would at home. While our data do not allow an assessment of this possibility, our data allowed us to at least rule out the first night effect on polysomnographically determined sleep variables, suggesting that any differences between laboratory and home sleep did not differentially affect S-TST compared to L-TST.

We have no objective information about the possible use of caffeine or other stimulants in the daytime prior to the PSG study in datasets 1 (São Paulo controls) and 2 (Treated OSA patients). Also, we have no objective information about sleep hygiene and habits in the days prior to the PSG study in dataset 2. This could be a limitation, but the strict absence of caffeine intake in the laboratory study of dataset 3 (which also included subjects refraining from caffeine intake during the 7 days before the laboratory experiment) as well as avoidance of irregular sleep hygiene and habits in datasets 1 and 3 (as evidenced by actigraphic recordings and/or questionnaires before the study)–while yielding consistent results—argues against caffeine and prior sleep as significant potential confounds in the study.

Another limitation is related to the different distributions and cut-off values of TST among datasets. In fact, more than half of the L-TST in dataset 1 (São Paulo controls) could be categorized as S-TST in datasets 2 (Treated OSA patients) and 3 (Healthy young adult controls). This difference could have come from differences in age distribution, culture, life style, disease history (dataset 2), and/or experimental protocol. While we cannot determine what specifically contributed to the observed differences in TST distributions, the consistent results *within* each dataset as well as across the combined datasets controlling for study and age indicates robustness and generalizability of the findings. Moreover, the consistent results of our additional analyses using the same TST cut point, for all datasets, with the data combined but statistically controlling for study and age, and also for each dataset separately, provide further evidence for the generalizability of the findings.

Dichotomizing subjects into two separate groups within each dataset might be a concern, as we observed that approximately one third of subjects in dataset 3 did not fall into the same group across the two nights. This is largely because the threshold value of the TST split was near the peak of the distributions (Fig 1), which maximizes the probability of crossing the threshold between nights. However, the correlation analyses of TST as a continuous variable against median duration of continuous sleep segments, which were not influenced by the dichotomization, corroborated our findings, indicating that they are robust.

Finally, natural sleep duration may be a function of dynamic changes hypothesized to occur in response to chronic sleep restriction/extension [6, 49, 50]. Subjects in dataset 3 (Healthy young adult controls) were required to maintain their habitual sleep schedule in the week prior to the laboratory measurements, but none of the studies we considered here measured or controlled sleep history in the preceding months or years. It has been posited that chronic sleep curtailment results in changes in the set point for sleep homeostasis, possibly mediated by adenosine receptor upregulation [30, 51], which could turn natural L-TST into habitual S-TST and vice versa while preserving normal homeostatic sleep regulation. This allostatic mechanism would result in differential vulnerability to cognitive impairment during periods of total sleep deprivation [6]. Further studies in moderate and extreme S-TST and L-TST measuring sleep homeostatic and objective performance responses to sleep deprivation could reveal to what extent allostatic changes induced by long-term sleep history may be involved in determining sleep duration.

## Conclusion

In conclusion, in both healthy controls and treated OSA patients, shorter total sleep times were associated with less sleep continuity. This study covered a wide range of naturally occurring sleep durations during normal sleep in healthy subjects and treated OSA patients (i.e., not just extremely short and long sleepers). Our results suggest that S-TST may differ from L-TST in processes underlying sleep continuity. This novel finding suggests a new perspective on physiological mechanisms of sleep continuity that could lead to individual differences in sleep duration.

## Supporting information

S1 File Hypnogram and age data for all subjects in all datasets. (XLS)

S2 File Additional analyses using the same cutoff for all datasets, with the data combined but statistically controlling for dataset and age, as well as analyzing each dataset separately, for sleep variables, sleep continuity, measures of sleepiness, continuity of sleep for whole-night hypnograms, continuity of sleep for the first 3 hours of the hypnograms, and continuity of wake for whole-night hypnograms. (PDF)

S3 File A bootstrap-based analysis that accounted for the number of segments contributed by each subjects for 1) continuity of sleep for whole-night hypnograms in each dataset, 2) continuity of sleep for the first 3 hours of the hypnograms in each dataset, 3) continuity of sleep and wake of the first and the second nights in dataset 3, and 4) continuity of wake for whole-night hypnograms in each dataset. (PDF)
