# Supplementary material for: Sleep continuity is positively correlated with sleep duration in laboratory nighttime sleep recordings
Source: PLoS One. 2017 Apr 10;12(4):e0175504. doi: 10.1371/journal.pone.0175504 (PMC5386280; doi:10.1371/journal.pone.0175504)
Supplement: S2 File — (PDF) [file pone.0175504.s002.pdf]

Supporting Information S2

Additional analyses were performed using the same TST cutoff for all datasets, with the data combined but statistically controlling for dataset and age, for 1) sleep variables, sleep continuity, and measures of sleepiness (Table S1), and 2) continuity of sleep for whole-night hypnograms, continuity of sleep for the first 3 hours of the hypnograms, and continuity of wake for whole-night hypnograms (Fig S1). The cutoff value was set to 434 min, which was the median TST of all subjects of all datasets. Prior to these analyses, age and BMI were compared between the groups. Mann-Whitney *U* tests were used to compare the S-TST and L-TST groups with regard to age and BMI. Analyses of covariance were used to compare S-TST versus L-TST on sleep variables, sleep continuity, and measures of sleepiness, including dataset and age as covariates. Cox proportional hazard models were used to compare S-TST versus L-TST on the survival curves, with dataset and age as covariates. Overall, the results were preserved ($P<0.05$), corroborating the findings of the analyses in the main text based on study-specific TST cutoffs. Moreover, no statistically significant results became non-significant when the analyses were repeated with the covariate for dataset removed, further supporting the findings.

Additional analyses were also performed using the same TST cutoff for all datasets but analyzing each dataset separately, for 1) age, BMI, sleep variables, sleep continuity, and measures of sleepiness (Table S2), 2) continuity of sleep for whole-night hypnograms (Fig S2), 3) continuity of sleep for the first 3 hours of the hypnograms (Fig S3; in line with the main analysis, this was not done for dataset 2 because of insufficient number of sleep segments), and 4) continuity of wake for whole-night hypnograms (Fig S4). The cutoff value was again set to 434 min. Mann-Whitney *U* tests were used to compare S-TST versus L-TST on age, BMI, sleep variables, sleep continuity, and measures of sleepiness. Generalized Wilcoxon tests were used to compare S-TST versus L-TST on the survival curves. Using the same TST cutoff among all datasets created unbalanced groups in each of the datasets separately and therefore limited statistical power for these specific analyses (in particular for the first night of dataset 3, which included only 4 subjects in the S-TST group based on a TST cutoff of 434 min). Nonetheless, the direction of the observed effects stayed the same as observed for the analyses in the main text.

Table S1: Characteristics of subjects, sleep variables, sleep continuity and measures of sleepiness using a single TST cutoff for all datasets combined.

	Combined Dataset	
	**S-TST**	**L-TST**
**Number of subjects**	139	140
**% Men**	54.0	60.0
**Age (years)**	37.5 ± 13.1	29.7 ± 8.1**
**Body mass index (kg/m^2)**	25.4 ± 5.3	27.2 ± 7.3*
Sleep structure		
**Time in Bed (min)**	412.8 ± 83.7	588.4 ± 29.3**
**Total Sleep Time (min)**	322.9 ± 70.3‡	514.5 ± 39.6‡
**Sleep Efficiency (%)**	79.1 ± 14.9	87.5 ± 5.7**
**Wakefulness After Sleep Onset (%)**	13.3 [6.6 – 22.3]	7.5 [4.8 – 11.8]**
**N1 (%)**	3.2 [1.9 – 5.3]	4.5 [3.3 – 7.0]
**N2 (%)**	44.8 [40.5 – 50.4]	49.5 [44.0 – 54.0]**
**N3 (%)**	19.6 [14.0 – 24.4]	14.3 [10.2 – 17.3]
**REM Sleep (%)**	15.3 [12.0 – 19.2]	22.9 [19.9 – 25.8]**
**Sleep Latency (min)**	10.5 [5.5 – 23.0]	17.0 [11.0 – 30.3]**
**REM Latency (min)**	91.5 [70.0 – 135.9]	72.5 [61.0 – 92.8]
**Number of Awakenings**	20.0 [14.0 – 27.0]	27.0 [21.0 – 33.0]
Sleep continuity		
**25th percentile for duration of continuous sleep segments (min)**	1.9 [1.0 – 4.5]	3.2 [2.0 – 6.4]
**Median duration of continuous sleep segments (min)**	7.0 [3.3 – 13.5]	11.6 [7.5 – 16.9]
**75th percentile for duration of continuous sleep segments (min)**	23.0 [14.7 – 33.9]	26.0 [19.8– 37.7]
Sleepiness		
**ESS**	5.0 [3.0 – 7.0]	5.0 [3.0 – 7.0]

Values are means ± SD or medians [25th percentile – 75th percentile]. ***P*<0.01, **P*<0.05 for difference between S-TST and L-TST. ‡No statistical tests were performed for TST as groups were selected on the basis of TST.

Table S2: Characteristics of subjects, sleep variables, sleep continuity and measures of sleepiness and fatigue for all datasets (groups analyzed separately but using the same TST cutoff among all datasets).

	Dataset 1		Dataset 2		Dataset 3			
					1st night		2nd night	
	S-TST	L-TST	S-TST	L-TST	S-TST	L-TST	S-TST	L-TST
**Number of subjects**	123	11	7	14	4	58	5	57
**% Men**	49.6	63.6	85.7	64.3	75.0	60.3	100.0	57.9
**Age (years)**	37.6 ± 13.1	29.5 ± 7.1*	49.7 ± 10.3	43.2 ± 13.2	24.8 ± 2.1	28.2 ± 5.6	28.2 ± 6.8	28.0 ± 5.4
**Body mass index (kg/m^2)**	24.9 ± 5.2	25.9 ± 3.4	32.0 ± 5.7	41.0 ± 12.2	27.7 ± 4.6	25.5 ± 4.5	24.9 ± 1.9	25.7 ± 4.6
Sleep structure								
**Time in Bed (min)**	392.8 ± 64.5	538.7 ± 38.2**	523.6 ± 37.6	533.9 ± 40.7	599.9 ± 0.3	599.8 ± 0.5	599.9 ± 0.2	599.8 ± 0.6
**Total Sleep Time (min)**	315.6 ± 67.5‡	474.8 ± 35.7‡	357.1 ± 98.0‡	469.4 ± 29.6‡	389.9 ± 14.6‡	524.4 ± 35.9‡	400.3 ± 33.0‡	523.1 ± 34.0‡
**Sleep Efficiency (%)**	80.7 ± 14.4	88.2 ± 4.3†	68.8 ± 20.1	88.2 ± 6.0**	65.0 ± 2.5	87.4 ± 6.0**	66.7 ± 5.5	87.2 ± 5.7**
**Wakefulness After Sleep Onset (%)**	12.4 [6.1 – 21.5]	10.7 [5.5 – 11.1]	16.9 [14.4 – 46.5]	8.8 [5.7 – 10.7]**	17.2 [4.0 – 31.5]	8.1 [5.4 – 12.5]	21.7 [14.5 – 26.0]	6.3 [4.6 – 10.5]*
**N1 (%)**	3.0 [1.7 – 4.5]	2.8 [2.4 – 4.0]	11.2 [10.6 – 13.2]	11.9 [9.8 – 14.2]	4.5 [3.6 – 5.2]	4.5 [3.5 – 6.4]	3.6 [2.7 – 5.0]	4.2 [3.1 – 6.3]
**N2 (%)**	44.8 [41.1 – 50.8]	45.2 [36.3 – 51.5]	48.5 [31.5 – 51.5]	50.5 [48.1 – 56.3]	42.1 [33.1 – 46.2]	49.0 [43.8 – 54.1]†	42.7 [39.3 – 46.6]	50.7 [45.0 – 54.0]*
**N3 (%)**	20.1 [15.3 – 24.5]	21.4 [17.8 – 25.2]	4.0 [3.1 – 7.1]	3.4 [0.5 – 12.5]	17.6 [14.4 – 22.6]	14.9 [10.9 – 17.3]	14.8 [9.0 – 22.4]	14.0 [10.9 – 16.0]
**REM Sleep (%)**	15.2 [11.7 – 19.3]	22.3 [18.5 – 25.8]**	14.2 [7.3 – 17.3]	21.6 [16.1 – 22.8]*	19.1 [16.9 – 22.5]	23.3 [19.3 – 25.5]	16.3 [15.2 – 19.9]	23.1 [20.6 – 26.4]**
**Sleep Latency (min)**	10.0 [4.6 – 21.0]	10.5 [5.4 – 20.3]	10.5 [7.1 – 53.8]	14.5 [8.0 – 21.5]	111.3 [22.3 – 200.0]	15.5 [10.5 – 27.0]*	51.0 [43.0 – 125.1]	20.0 [14.4 – 40.6]**
**REM Latency (min)**	90.5 [71.4 – 135.9]	71.5 [61.3 – 95.0]†	133.0 [87.1 – 149.3]	85.0 [62.5 – 186.5]	80.5 [63.8 – 101.3]	71.8 [61.5 – 100.5]	63.5 [62.1 – 110.9]	72.5 [60.4 – 84.1]
**Number of Awakenings**	20.0 [14.0 – 26.0]	25.0 [20.0 – 30.8]†	30.0 [23.5 – 41.8]	19.0 [18.0 – 33.0]	20.0 [19.0 – 24.5]	27.0 [22.0 – 35.0]	25.0 [21.5 – 35.5]	28.0 [24.0 – 33.0]

Sleep continuity								
**25th percentile for duration of continuous sleep segments (min)**	1.9 [1.0 – 3.4]	3.0 [2.3 – 6.3]	1.0 [0.6 – 1.4]	1.5 [1.0 – 4.8]	4.7 [4.1 – 6.5]	3.0 [2.0 – 6.3]	3.5 [1.5 – 5.0]	3.8 [2.2 – 6.7]
**Median duration of continuous sleep segments (min)**	6.5 [3.3 – 13.9]	11.5 [9.0 – 17.9]†	4.8 [2.3 – 10.8]	9.0 [5.5 – 19.8]	11.5 [9.5 – 13.6]	12.1 [7.5 – 19.5]	7.0 [5.6 – 8.6]	11.3 [8.4 – 14.1]*
**75th percentile for duration of continuous sleep segments (min)**	24.0 [14.8 – 35.0]	31.3 [22.6– 35.6]	16.3 [10.8 – 36.2]	32.3 [19.3 – 47.0]	25.8 [17.0 – 31.6]	24.4 [19.8 – 38.0]	17.5 [12.7 – 20.1]	25.5 [19.5 – 34.2]*
Sleepiness and fatigue								
**ESS**	5.0 [3.0 – 7.0]	4.0 [3.0 – 8.0]	6.0 [5.0 – 8.0]	6.5 [4.0 – 9.0]	5.0 [3.5 – 6.0]	5.0 [3.0 – 7.0]	7.0 [3.8 – 7.3]	5.0 [3.0 – 6.3]
**CFS**	1.0 [0.0 – 3.0]	1.5 [1.0 – 3.0]						
**PVT lapses**			8.5 [4.7 – 10.5]	9.1 [5.8 – 13.1]	1.2 [0.7 – 1.9]	0.5 [0.2 – 1.2]	0.5 [0.5 – 2.2]	0.5 [0.2 – 1.3]
**MSLT (min)**			2.2 [1.3 – 4.3]	2.5 [1.6 – 8.5]				

Values are means ± SD or medians [25th percentile – 75th percentile]. **$P<0.01$, *$P<0.05$, †$P<0.10$ for difference between S-TST and L-TST. ‡No statistical tests were performed for TST as groups were selected on the basis of TST.

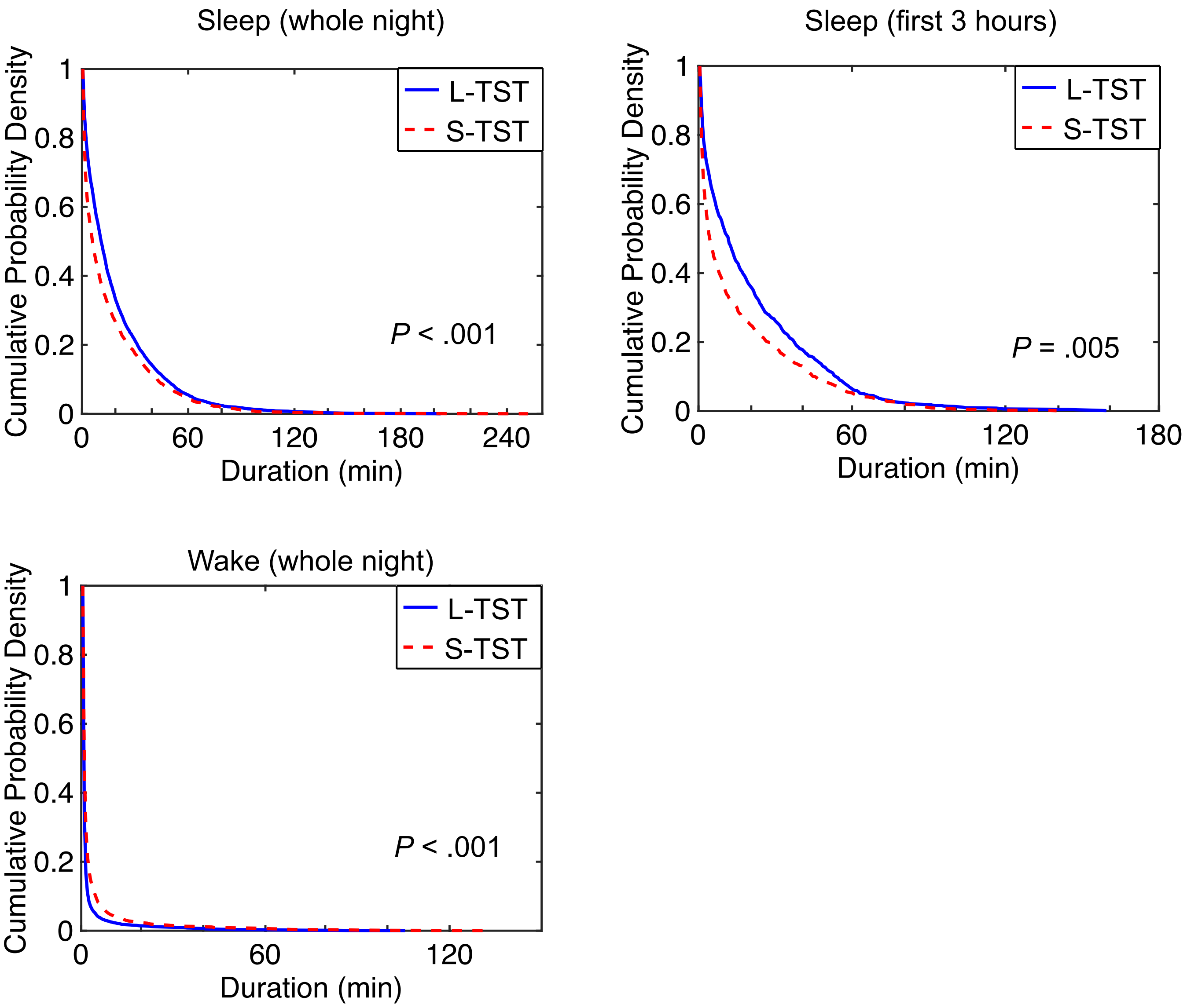

Fig S1. Continuity of sleep for whole-night hypnograms and the first 3 hours of the hypnograms and for continuity of wake for whole-night hypnograms using a single TST cutoff for all datasets combined. Figure details are the same as for Figs 2, 3 and 5 in the main text.

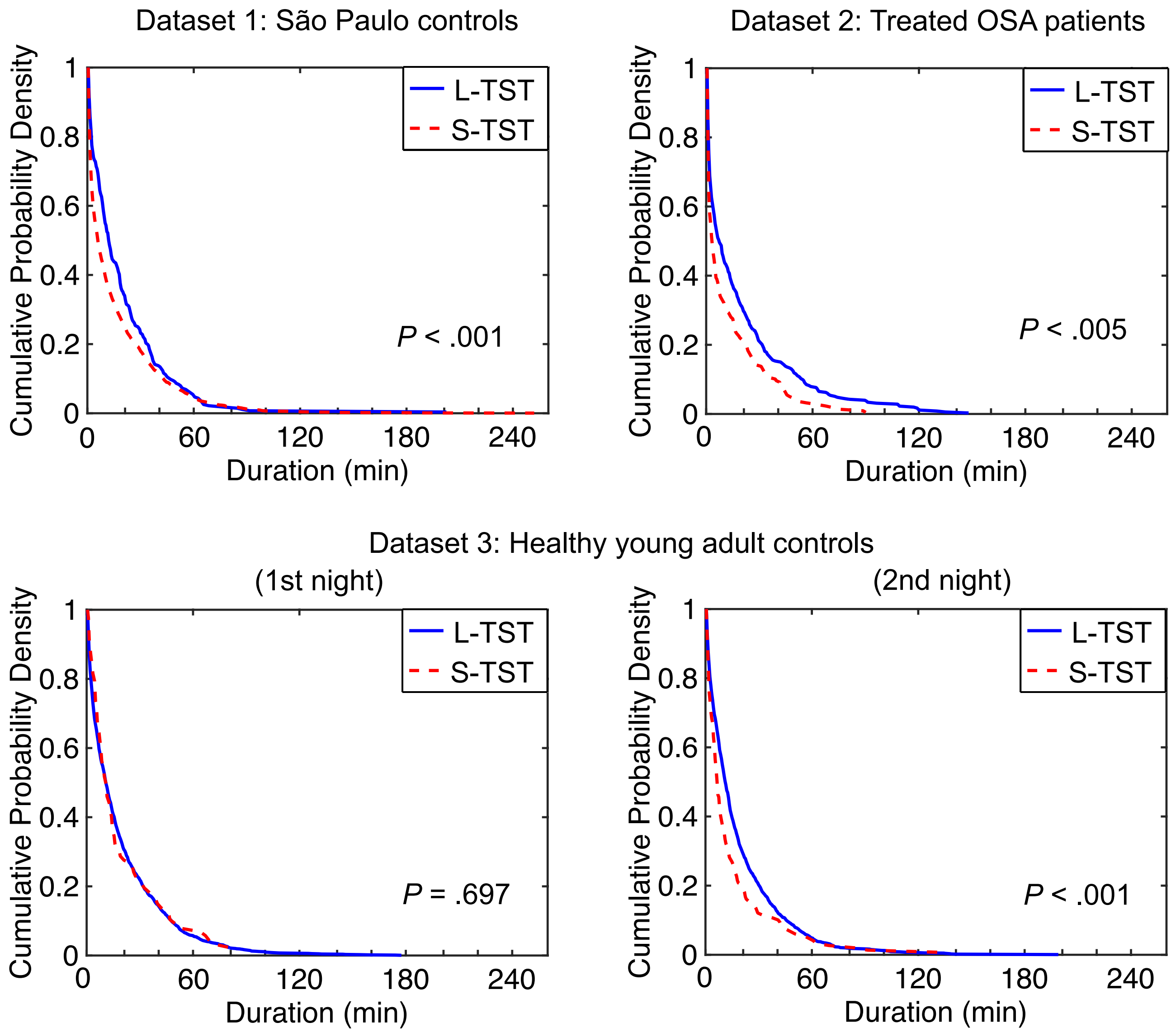

Fig S2. Continuity of sleep for whole-night hypnograms in each of the datasets analyzed separately but using the same TST cutoff among all datasets. Figure details are the same as for Fig 2 in the main text.

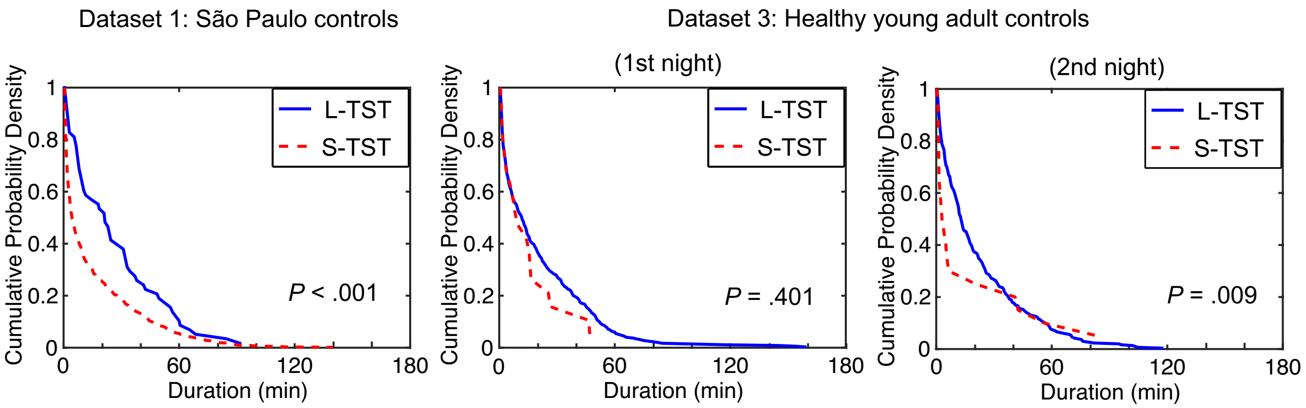

Fig S3. Continuity of sleep for the first 3 hours of the hypnograms in each of the datasets analyzed separately but using the same TST cutoff among all datasets. Figure details are the same as for Fig 3 in the main text.

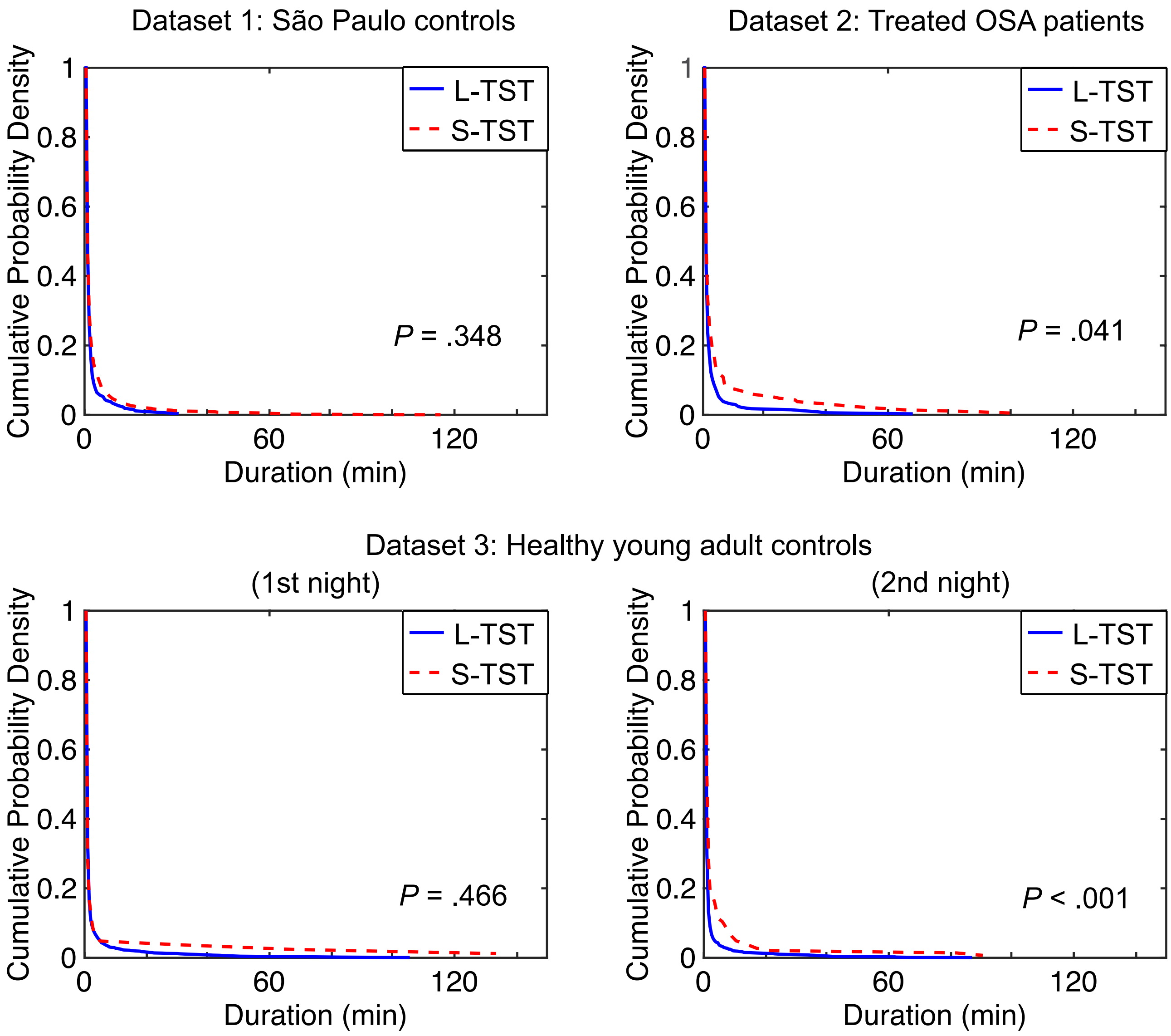

Fig S4. Continuity of wake for whole-night hypnograms in each of the datasets analyzed separately but using the same TST cutoff among all datasets. Figure details are the same as for Fig 5 in the main text.
