# Supplementary material for: Sleep continuity is positively correlated with sleep duration in laboratory nighttime sleep recordings
Source: PLoS One. 2017 Apr 10;12(4):e0175504. doi: 10.1371/journal.pone.0175504 (PMC5386280; doi:10.1371/journal.pone.0175504)
Supplement: S3 File — (PDF) [file pone.0175504.s003.pdf]

Supporting Information S3

A bootstrap-based analysis that accounted for the number of segments contributed by each subjects was performed for 1) continuity of sleep for whole-night hypnograms in each dataset, 2) continuity of sleep for the first 3 hours of the hypnograms in each dataset, 3) continuity of sleep and wake of the first and the second nights in dataset 3, and 4) continuity of wake for whole-night hypnograms in each dataset. Striking a balance between equivalence among subjects and retaining a sufficient number of data points, an equal number of segments was sampled from subjects who had segments more than median number of segments in each dataset, whereas for the rest of the subjects all segments were kept in the analysis. Generalized Wilcoxon tests were used to compare S-TST versus L-TST on the survival curves derived from the sampling procedure. This procedure was repeated 1,000 times. At each step of the iteration, a p value was estimated from the history of prior p values, yielding an increasingly stable result as the number of iterations increases. The final results of the analyses are shown in Figs S5–S8. They corroborate the findings of the analyses in the main text, which were performed on the full data set with all segments included.

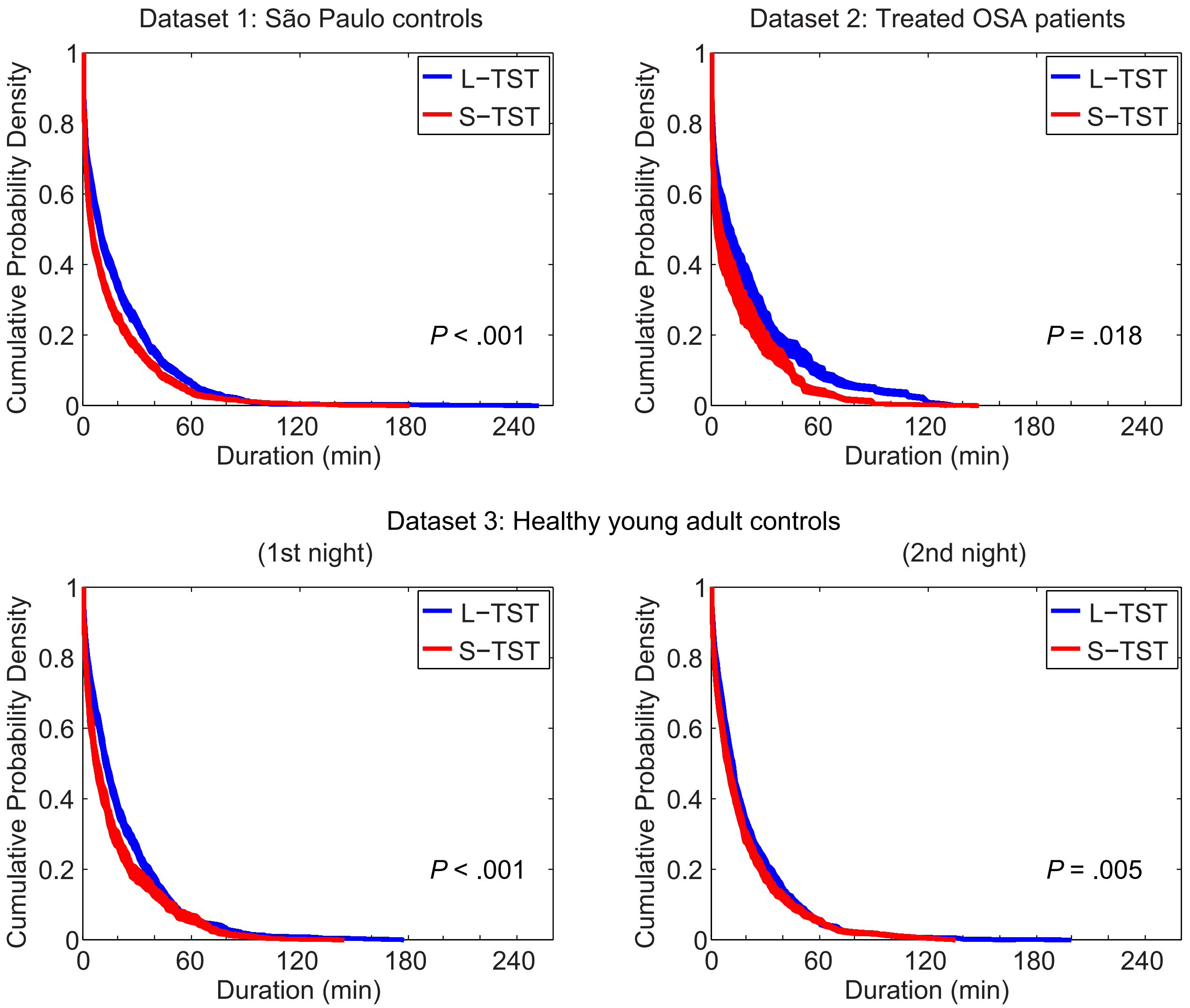

Fig S5. Continuity of sleep for whole-night hypnograms in each dataset, derived from the bootstrap analysis. Figure details are the same as for Fig 2 in the main text. Line thickness reflects variability among the bootstraps (all 1,000 curves are shown for each group).

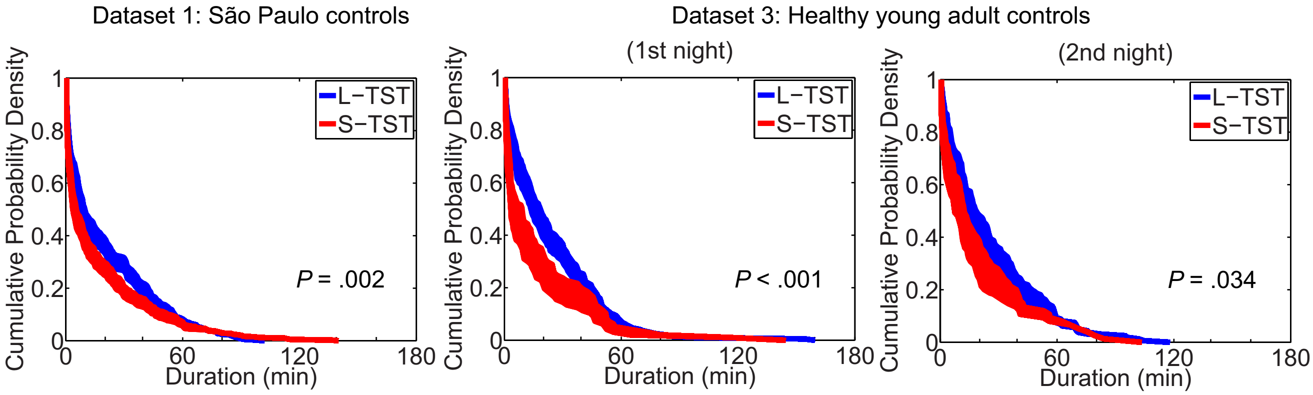

Fig S6. Continuity of sleep for the first 3 hours of the hypnograms in each dataset, derived from the bootstrap analysis. Figure details are the same as for Fig 3 in the main text. Line thickness reflects variability among the bootstraps (all 1,000 curves are shown for each group).

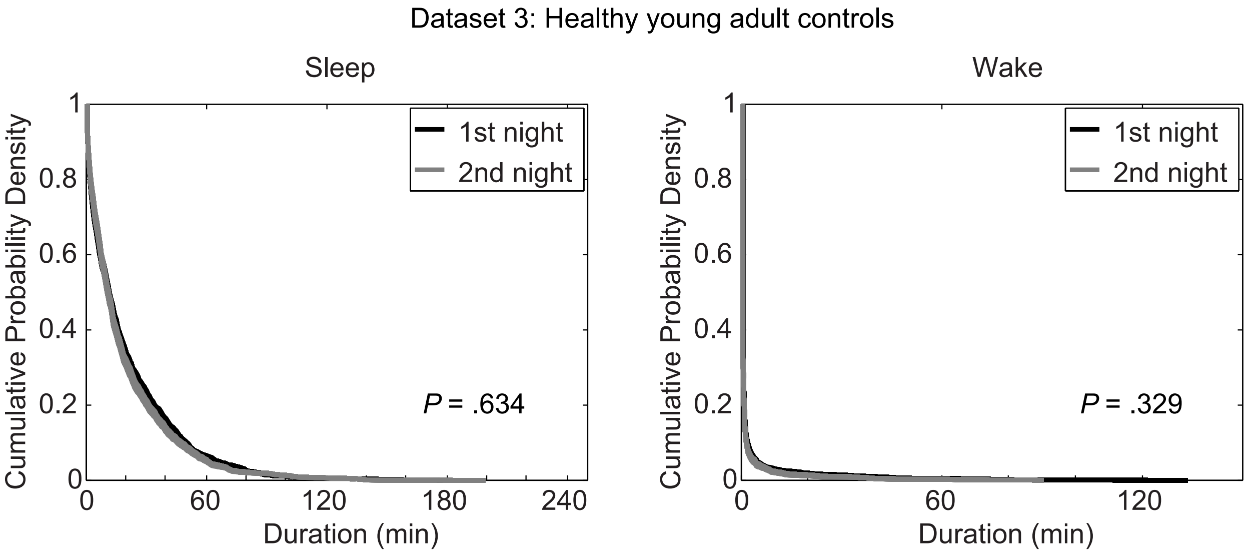

Fig S7. Continuity of sleep and wake of the first and the second nights in dataset 3, derived from the bootstrap analysis. Figure details are the same as for Fig 4 in the main text. Line thickness reflects variability among the bootstraps (all 1,000 curves are shown for each group).

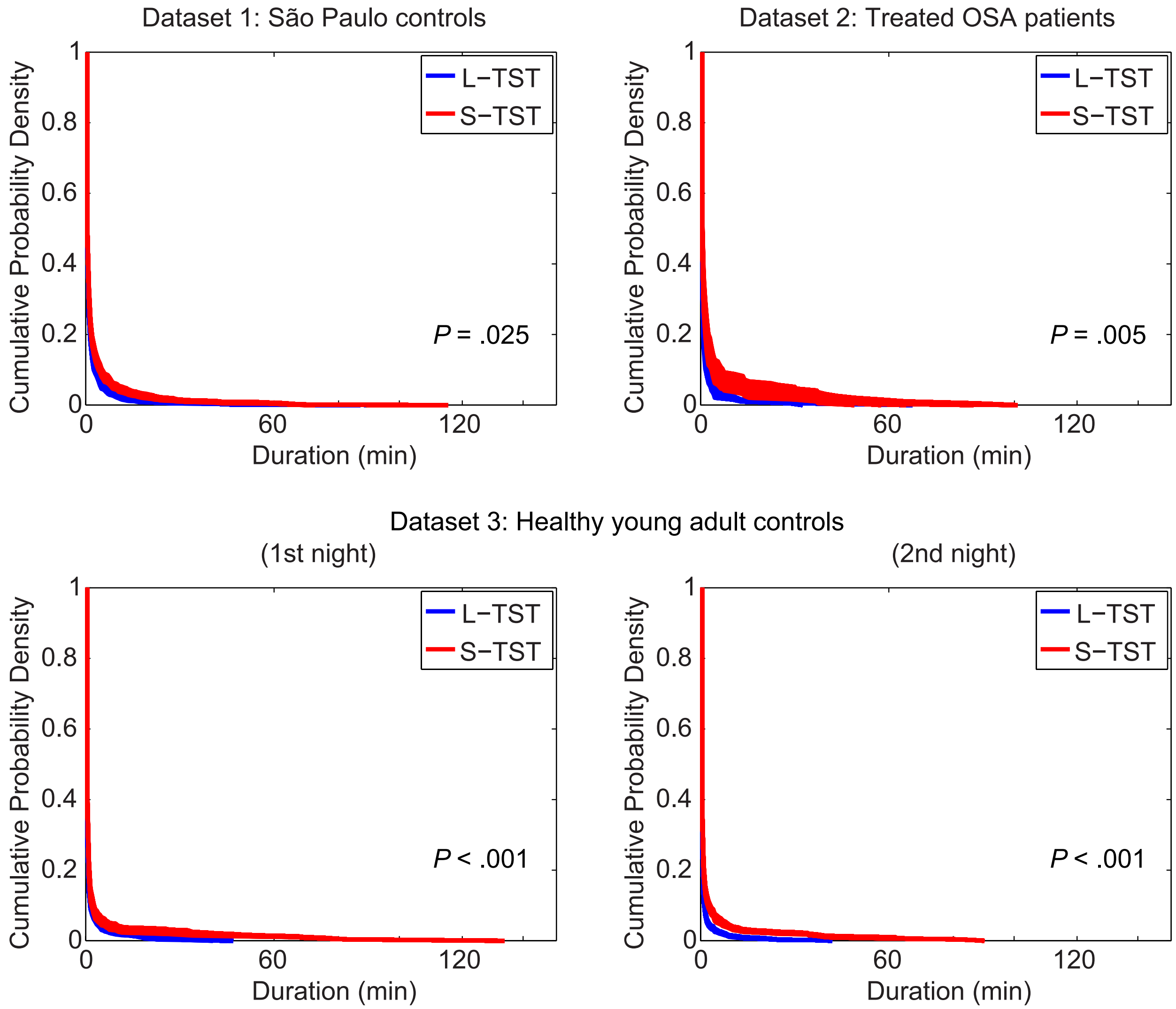

Fig S8. Continuity of wake for whole-night hypnograms in each dataset, derived from a bootstrap-based analysis. Figure details are the same as for Fig 5 in the main text. Line thickness reflects variability among the bootstraps (all 1,000 curves are shown for each group).
